# Querying the public databases for sequences using complex keywords contained in the feature lines

**DOI:** 10.1186/1471-2105-7-45

**Published:** 2006-01-27

**Authors:** Olivier Croce, Michaël Lamarre, Richard Christen

**Affiliations:** 1 Laboratoire de Biologie Virtuelle, UMR 6543, CNRS & University of Nice Sophia-Antipolis, Centre de Biochimie, Parc Valrose, Nice, F06108, France

## Abstract

**Background:**

High throughput technologies often require the retrieval of large data sets of sequences. Retrieval of EMBL or GenBank entries using keywords is easy using tools such as ACNUC, Entrez or SRS, but has some limitations, in particular when querying with complex keywords.

**Results:**

We show that Entrez has severe limitations with respect to retrieving subsequences. SRS works well with simple keywords but not with keywords composed of several terms, and has problems with complex queries. ACNUC works well, but does not allow precise queries in the Feature qualifiers. We developed specific Perl scripts to precisely retrieve subsequences as defined by complex descriptors in the Features qualifiers of the EMBL entries. We improved parts of the bioPerl library to allow parsing of large data files, and we embedded these scripts in a user friendly interface (OS independent) for easy use.

**Conclusion:**

Although not as fast as the public tools that use prebuilt indexes, parsing the complete entries using a script is often necessary in order to retrieve the exact data searched for. Embedding in a user friendly interface allows biologists to use the scripts, which can easily be modified, if necessary, by bioinformaticians for unforeseen needs.

## Background

The quantity of biological information available in the public databases is now very large and doubling nearly every year [1]. Projects involving high throughput data from approaches such as the new transcriptomic or genomic technologies require large quantities of data to be dealt with. For example, in order to design a DNA chip for bacterial identification, one may want to retrieve every available sequence for a universal gene such as the 16S rRNA gene (nearly 200,000 sequences expected by the end of year 2005).

Retrieval of such sequences is not trivial. Retrieval by sequence similarity is not feasible, since some of these genes are very variable (ITS regions for example). Also it is difficult, if not impossible, to determine a cutoff level in order to exclude non homologous gene sequences. Blast [2] for example allows a cutoff according to the "E value", while Exonerate [3] also allows a cutoff on percentage of similarity. The "E value" cutoff depends on the database size that changes every day. The percentage of similarity is better, but for divergent ubiquitous genes, experiments show that some homologous sequences are not retrieved while non homologous sequences are retrieved. Importantly, a similarity search may return only parts of sequences, and it is difficult to automatically retrieve entire sequences.

The best solution seems to be a retrieval based on keywords that describe the particular feature to retrieve. This is however not always easy. Keywords used to describe a particular feature may be diverse and sometimes mis-spelled. The entire list of keywords can be retrieved (with ACNUC for example), parsed and painfully analysed to build a complete list of keywords. This task is more difficult with queries composed of complex keywords containing several words or numbers. Popular tools such as ACNUC [4], Entrez [5] or SRS [6] have been designed for the purpose of querying with keywords, but we show in this paper that they should be used with care and caution and that they still have flaws for precisely retrieving sequences according to a complex keyword. This is known by bioinformaticians working in this field who often use their own parsers to directly analyse entries, and in part led to the development of the bio-(Java, Perl, Python) libraries to name only the most commonly used. However, these wide purpose libraries have problems in particular for parsing large data files. We have developed a derivative of the bioPerl library and embedded it in a user friendly interface that makes it possible to easily define complex keywords, the fields to search for and to define the order for searching in these fields. Subsequences are automatically extracted and stored as files or directly in a "biosql" standard relational database for easy management.

## Implementation (EmblEx)

EmblEx is composed of three parts: i) a series of perl scripts, ii) a MySQL database and iii) a user-friendly graphic interface for queries through a web browser.

Emblex searches in ASCII files downloaded from a ftp server such as that of EBI [7]. Their URL can be stored by EmblEx, and files can be automatically downloaded and uncompressed with EmblEx, an internal routine allows to check if this file had been updated after the previous download.

Users can then define complex keywords (or regular expressions) to be searched for, the feature keys to search in, in which order, and the output format. Subsequences and related information are then extracted for the specific feature starting from the first feature as indicated in EmblEx. The available BioPerl module for extracting data from EMBL files was found to be too slow and replaced by a dedicated extractor. Complex feature entries, such as those containing the join() and complement() operators, or those using the "acc_no:xxx..xxx" syntax, where one feature calls upon a fragment of another sequence, are properly processed by EmblEx. Possible outputs are ASCII, HTML and within a MySQL database in BioSQL specification. Indeed, BioSQL is an emerging standard for biological data [8]. Its substantial advantage is the large number of scripts already developed within the BioPerl, BioPython, BioJava communities. The bioperl-db script used to load the database, originally developed for Linux systems only [9] was ported to MS Windows.

Because it may be difficult for many biologists to use scripts from the command line, we imbedded our scripts within a user friendly interface. EmblEx's interface was developed in HTML in order to be compatible with any systems and web browser.

Files available for download from our server [10] include every necessary dependency and are easily installed on every system. An online version is also available on our web server (no installation is required but some features have been disabled such as BioSQL database and update module).

## Results

We became aware of a problem when we were not able to retrieve some sequences with SRS, yet we knew they were in the EMBL database. For the purpose of the demonstration, we will in this paper focus on the retrieval of nuclear ribosomal intervening sequences (ITS, a short domain located between the small and large ribosomal RNA subunits, transcribed) which are widely used for the purpose of bacterial and protozoan identification [11-23]. We will restrict our experiment to the keyword "internal transcribed spacer 1", although we know that this region is also described using alternate keywords (Table 1)

**Table 1:** Keyword variations and mis-spelling.

ITS1, INTERNAL TRANSCIBED SPACER 1	1 seqs.
ITS1, INTERNAL TRANSCRIBED SPACER 1	22 seqs.
INTERNAL TRANSCRIBED SPACER 1, ITS1	32 seqs.
INTERNAL TRANSCRIBED SPACER 1 (ITS1)	166 seqs.
INTERNAL TRANSCRIBED SPACER 1	70,663 seqs.
INTERNAL TRANSCRIBED SPACER I	15 seqs.
INTERNAL TRANSCRIBED SPACER 1 OF RIBOSOM	9 seqs.
INTERNAL TRANSCRIBED SPACER 1; ITS 1	4 seqs.
INTERNAL TRANSCRIBED SPACER 1 (ITS1) OF	2 seqs.
INTERNAL TRANSCRIBED SPACER ITS1 OF RIBO	1 seqs.
INTERNAL TRANSCRIBED SPACER 1 (ITS1) OR	1 seqs.
INTERNAL TRANSCRIBED SPACER 1	2 seqs.
INTERNAL TRANSCRIBED SPACER 1; ITS1	65 seqs.
INTERNAL TRANSCRIBED SPACER 1; ITS-1	65 seqs.
INTERNAL TRANSCRIBED SPACER ITS1	22 seqs.
INTERNAL TRANSCRIBED SPACER 1 ITS1	45 seqs.
INTERNAL TRANSCRIBED SPACER 1; 5.8S RIBO	29 seqs.
INTERNAL TRANSCRIBED SPACER 1, 5.8S RIBO	65 seqs.
INTERNAL TRANSCRIBED SPACER REGION 1	35 seqs.
INTERNAL TRANSCRIBED SPACER REGION 2	35 seqs.
INTERNAL TRANSRIBED SPACER 1	1 seqs.
INTERNAL TRANSCIBED SPACER 1	6 seqs.
CONTAINS INTERNAL TRANSCRIBED SPACER 1,	368 seqs.
INTERNAL TRANSRCIBED SPACER 1	10 seqs.
RIBOSOMAL INTERNAL TRANSCRIBED SPACER 1	42 seqs.
INTERNAL TRANCSRIBED SPACER 1	1 seqs.
CONTAINS INTERNAL TRANSCRIBED SPACER 1 A	1 seqs.
INTERNAL TRANSCRIBED SPACER 1 AND 5.8S R	45 seqs.
INTERNAL TRNSCRIBED SPACER 1	1 seqs.
INTERNAL TRANSCRIBER SPACER 1	1 seqs.
INTERNAL TRANSCRIBED SPACER 1 TYPE I	1 seqs.
INTERNAL TRANSCRIBED SPACER 1 TYPE II	1 seqs.
INTERNAL TRANCRIBED SPACER 1	1 seqs.
INTERNAL TRANSCRIBED SPACER 1 (ITS1) REG	12 seqs.
ITS1 INTERNAL TRANSCRIBED 2	1 seqs.
INTERNAL TRANSCRIBED SPACER (ITS1)	16 seqs.
INTERNAL TRANSCRIBED SPAVER 1	119 seqs.

ACNUC was used to retrieve in the entire EMBL release 84 (all organisms), existing variations in features annoted with "internal transcribed spacer 1" (responses are truncated on the right). We can observe large variations as well as mis-spelling. For confirmation purpose, we queried EMBL release 84 for keyword "ITS1". ACNUC returned 16,392 entries while SRS at EBI returned 16,135 entries, due to problems with spaces and extra characters such as commas.

For simplicity (the whole public database changes every day), comparative queries were done on EMBL release 84 (September 2005). We wanted to search only within features: "misc_feature", "misc_rna", "gene", "rrna", "intron" and "source", to exclude retrieval of amplification primers for example. If the internal transcribed spacer 1 is described both in the "source" and "misc_rna" Feature keys, we wanted to extract the subsequence as defined by the boundaries indicated in the "misc_rna" qualifier. Finally, for simplicity, when an entry contained more than one ITS domain, only the parent entry accession number was considered (link to parent database with SRS, scripting with python for the other tools).

### 1/Entrez

Among biologists, Entrez is probably the most popular tool to retrieve sequences (and other data). It is poorly adapted for our purpose as it inefficiently scans the feature lines and cannot extract subsequences. Without subsequence extraction according to the boundaries described in the features lines, we would have to use a dedicated parser anyway.

We tried "Fungi" [Organism] AND srcdb_genbank [PROP] AND "internal transcribed spacer 1" [Feature key] AND gene_in_genomic [PROP].

This returned 28,592 entries (note that it may change everyday as Entrez does not allow a query on the release or update databases).

### 2/SRS

SRS is a particularly powerful tool. Using the extended web form, it is possible to easily build powerful queries. The internal getz langage can also be used either from the command line or using the "Result" page. Using the getz alternative, it is possible to use regular expressions. Several queries were tried (in its "Result" section, SRS reformulates these queries differently, but they seem to run faster as written below).

[emblrelease-Division:fun] > ([emblrelease-FtKey:gene|intron|misc_feature|misc_rna|rrna|source] & [embl-FtDescription:internal transcribed spacer 1])

This query returned an "*Error: No entries found *" message (but see below). Replacing "internal transcribed spacer 1" by "internal?transcribed?spacer?1" returned the same message. We then used the wild card "*" instead of "?" and obtained the same message.

We had to use the AND ("&") operator to obtain a response.

Q1: [emblrelease-Division:fun] > ([emblrelease-FtKey:gene|intron|misc_feature|misc_rna|rrna|source] & [emblrelease-FtDescription:internal&transcribed&spacer&1])

This query took about two minutes and returned 33,767 entries and was reformulated by SRS as shown below:

Query "([emblrelease-Division:fun] > (((((([emblrelease-FtKey:gene] | [emblrelease-FtKey:intron]) | [emblrelease-FtKey:misc_feature]) | [emblrelease-FtKey:misc_rna]) | [emblrelease-FtKey:rrna]) | [emblrelease-FtKey:source]) & ((([emblrelease-FtDescription:internal] & [emblrelease-FtDescription:transcribed]) & [emblrelease-FtDescription:spacer]) & [emblrelease-FtDescription:1]))).

Since we suspected that more entries could be obtained we also tried:

Q2: [emblrelease-Division:fun] > ([emblrelease-FtKey:gene|intron|misc_feature|misc_rna|rrna|source] & [emblrelease-FtDescription:internal&transcribed&spacer&1*])

After a very long time, this query returned an error message in the form of an error of memory allocation "*Error: Insufficient memory – error during malloc *". The query was tried on week end, when the EBI server is less busy but it returned the same message; other servers offering SRS [24] also returned the same error message. When asked, EBI people answered that too many data had to be treated for the memory size of their server.

We then tried to use a regular expression, which took a very long time and returned an error message:

Q3: [emblrelease-Division:fun] > ([emblrelease-FtKey:gene|intron|misc_feature|misc_rna|rrna|source] & [emblrelease-FtDescription:/internal transcribed spacer 1/])

The following query also returned an error message.

[emblrelease-Division:fun] > ([emblrelease-FtKey:gene|intron|misc_feature|misc_rna|rrna|source] & [emblrelease-FtDescription:/internal transcribed spacer/]

Finally a query with /spacer/found 62,863 entries, a query with "/internal/" found 60,637 entries and queries with "/internal spacer/" or "/internal\sspacer/" both returned a message "*Error: No entries found *" demonstrating that (on ech public server tried) SRS has a problem with spaces, both in the extended form and regular expressions (see the discussion).

The same queries were done on the Infobiogen server, the same day (September 16th, 2005). These queries returned very different numbers of entries (Table 2). Parse and analysis of the results (see below) showed a potential problem with SRS at Infobiogen. When asked, the Infobiogen people answered, acknowledging a problem with the SRS indexes. SRS did not return an error message, but the entries obtained were globally wrong; among the 15,108 response unique to SRS at Infobiogen (see below) we found for example accession number AB011433, which refers to a protein coding sequence.

**Table 2:** Compararison of results obtained using similar queries and different tools or servers.

	total	SRS ebi	EmblEx	SRS infobiogen	ACNUC
SRS ebi	33,758		64	24,520	71
EmblEx	33,697	2		24,495	7
SRS infobiogen	24,346	15,108	15,145		15,145
ACNUC	33,692	5	3	24,491	

The first column indicates how many different accession numbers were returned in each case. Other columns indicate how many of these accession numbers were not retrieved by the other procedures.

### 3/ACNUC

For querying with ACNUC we used the " raa_query" client (available on the pbil server [25]). When we started this analysis, we found that ACNUC had a bug that did not allow it to find complex keywords spanning two lines. When informed, ACNUC people corrected this bug, and the present queries were done on ACNUC after correction (September 16th, 2005).

A first list was built with command se-sp = fungi, a second list was built with command se-o = nuclear; intersection of the two lists returned 866,080 results. The -o option is necessary as ACNUC stores as "fungi" the mitochondrial and nucleomorph sequences (no chloroplastic sequence known in fungi). Intersection with "k = division fun" was then necessary in order to exclude sequences from the "ENV" division. Finally, it would have been possible to use intersections with successive queries in the form " k = misc_rna" in order to include only the proper FT keys, and then to proceed to an union of the lists, but this can be tedious and we have no direct possibility to choose among the fields "source" and "misc_rna" to extract the proper subsequence according to the boundaries described in the most specific feature.

A recent implementation in ACNUC allows to scan specific lines such as the FT lines. This is much slower, as ACNUC cannot use its index tables, but allows a more precise selection. The final list was queried using command mo -7 and searching FT lines for "internal transcribed spacer 1". This query took about three minutes and returned 33, 692 entries. ACNUC did not find seven entries found by EmblEx (X93976, X93980, X93987, Z48813, Z48817, Z48818, Z48819), due to a problem discussed below.

### 4/Extraction using a dedicated script

We used EmblEx to parse the EMBL fun.dat file, which contains all of the fungi sequences (release 84), for the presence of "internal transcribed spacer 1" in the following features: misc_feature, misc_rna, gene, rrna, intron or source, in that order. This query took seven minutes and returned 33,696 entries.

### 5/Analysis

A python script allowed to parse the results and to analyse which accession numbers were retrieved by one of the tools and not retrieved by another one (Table 2). As shown in this table, ACNUC, SRS on EBI and our Perl script had similar, but different results. As indicated above, SRS on Infobiogen returned wrong results because of index problems. Next we looked for entries retrieved only by a single tool (Table 3). SRS at EBI had 27 such entries, EmblEx none and ACNUC 3.

**Table 3:** Unique answers.

SRS ebi	27
EmblEx	0
SRS infobiogen	15,108
ACNUC	3

This table shows how many accession numbers are unique to a query (retrieved only by a single tool).

The 64 SRS entries not found by EmblEx (including the 27 entries unique to SRS EBI) were manually scanned and results indicated in table 4. All of the entries contain the keywords searched for, but either with other words in between or in the wrong order. The entries specific to ACNUC were also examined. The complex keyword was easily found, but in wrong features ("snorna" or "precursor_rna" for example).

**Table 4:** Annotations of the 64 responses of SRS not found by EmblEx.

53	**internal transcribed spacer **region **1**
7	**Internal transcribed spacer (ITS) 1 region**
1	**transcribed spacer 1**, 5.8S ribosomal RNA, **internal...**
2	RNA, **transcribed spacer 1**, 5.8S ribosomal RNA, **internal**...
1	contains *nternal ***transcribed spacer 1**, 5.8S ribosomal RNA, **internal...**

The seven entries found by EmblEx and not by ACNUC corresponded to entries having "internal transcribed spacer 1" in the proper feature, but that had been submitted as "unknown" organism, latter corrected to "fungi" but without any change of the DT "last updated" field. Since ACNUC updates its databases only for entries that are recorded as changed since last update, these entries could not be identified as of fungal origin. ACNUC people, when informed answered that they would correct this problem in the near future.

## Conclusion

Analyses of responses found by SRS, Entrez, ACNUC and a dedicated script showed:

1/Entrez capabilities for retrieving the proper data set are limited as specific FT lines cannot be assigned. Also, subsequences are not automatically extracted. Both limitations render Entrez unfit for the purpose sought.

2/SRS retrieved some entries not present in EmblEx, all were false positives that mostly consisted of sub-entries annotated as "internal transcribed spacer region 1". Although in this case the answer is right, this is not a proper answer and could lead in other cases to unwanted sequences. More important, the day of the final demonstration query, SRS at Infobiogen returned a wrong dataset, with no error message, due to indexing problems. EmblEx retrieved some entries that SRS missed, mostly due to a problem with the presence of white spaces and parentheses, as in accession numbers AB176462 & AB176463, with a misc_rna feature containing "contains internal transcribed spacer 1,2, 5.8S ribosomal RNA". SRS could be used to index phrases, but the administrators are (usually) not doing it, one reason probably is that using the exact phrase is only appropriate when one already knows that phrase. Most of the time users have much fuzzier queries – at least until they have found what they want and can start to refine it. Finally, SRS showed problems when the amount of memory necessary to run the queries turned out to be too big. On the EBI server for example, the Q1 query indicated above but run on Embl (Emblrelease + Emblnew) returned a memory allocation error message, which is presumably not due to SRS per se but to the available memory of the hardware used to run SRS.

3/ACNUC had no problem of memory of any sort and was fast as long as scanning the FT lines was not required. It retrieved entries not found by the Perl script; they corresponded to keywords for other features (such as "snorna" and "precursor_rna"), since we did not use a search in specific features for reasons mentionned above.

4/If the data obtained are of importance, it is safer to query different servers and compare the results obtained.

5/Extensive analyses of results provided by the public tools can detect subtle bugs or problems due to the semi-structured form of the EMBL/GenBank entries.

In a recent work, D'Addabbo and coworkers [26] also noticed a problem in extracting data from feature definition with SRS; their solution (GeneRecords) was to build extraction procedures to import GenBank entries in a FileMaker^© ^database. Compared to GeneRecords, EmblEx makes use of non proprietary software and runs under every OS (developed under Linux and MS-Windows for compatibility). It also renders it possible to import directly in the BioSQL format.

A script is obviously not as fast as ACNUC or SRS, since no index tables are used, but is more flexible and precise. It is however possible to first use SRS or ACNUC to extract a large number of entries and then run EmblEx on these entries for a final finely tuned analysis, combining speed of extraction with precision. In particular EmblEx allows users to specify a search order in the annotations, making sure for example that if both "ITS1" and "ITS2" are in the "source" field, and "ITS1" in the "misc_rna" field, only the latter sequence will be properly extracted (which could be done using ACNUC and a dedicated script).

In conclusion, the advantages of EmblEx are in particular i) easy modifications of the code for a particular use, ii) use of the BioSQL standard, therefore allowing subsequent use of public routines (BioJava, BioPython and BioPerl), iii) very precise queries through a graphical interface or the command line and, iv) simple local use or access to a distant server through the web.

More details can be found in the html documentation provided with EmblEx.

## Availability and requirements

• **Project name: **EmblEx

• **Project home page: **

• **Operating system(s): **Windows, Unix

• **Programming language: **Perl, HTML

• **Other requirements: **Apache 1.x or higher, MySQL 3.x or higher

• **License: **Open Source 

## Authors' contributions

ML and OC implemented the software (Perl scripts) and the graphical user interface, and were the primary authors of the manuscript. RC participated in design and coordination and wrote the Python scripts. All authors read and approved the final manuscript.
